# Generalized Pustular Psoriasis as a Systemic Inflammatory Disease: Experience With 38 Japanese Cases Over 15 Years at a Single Institution

**DOI:** 10.7759/cureus.88108

**Published:** 2025-07-16

**Authors:** Nobuyuki Kikuchi, Misaki Kusano, Toshiyuki Yamamoto

**Affiliations:** 1 Dermatology, Fukushima Medical University, Fukushima, JPN

**Keywords:** clinical data, comorbidities, il-36, pustular psoriasis, s100a, systemic inflammation

## Abstract

Pustular psoriasis is a rare subtype of psoriasis, classified into two broad forms: generalized and localized. In the present study, we retrospectively analyzed 41 cases of pustular psoriasis over a 15-year period at a single institution. There were 38 cases of generalized and three cases of localized pustular psoriasis. Among the 38 patients with generalized pustular psoriasis (GPP), 15 (39.5%) had a prior history of psoriasis, while the remaining patients developed GPP de novo. Eighteen patients were former or current smokers, and arthritis was observed in 12 patients. Four patients, including one with Turner syndrome, developed GPP in childhood. Episodes of generalized pustulation occurred once in seven patients, once in three patients, twice in three patients, and three times in one patient. The triggers of pustulation were upper respiratory infection (n=2), pregnancy (n=2), withdrawal of methotrexate (n=1), and paradoxical induction by infliximab (n=2). The comorbidities were hypertension (n=10), diabetes mellitus (n=6), dyslipidemia (n=2), hyperuricemia (n=2), chronic kidney disease (n=2), ischemic heart disease (n=4), Crohn’s disease (n=3), and acute respiratory distress syndrome (n=1). Nail involvement was observed in 21 patients. Treatment included etretinate in 20 patients, cyclosporine in nine, methotrexate in two, and apremilast in one. Biologics were used in 14 patients: infliximab in eight, secukinumab in six, adalimumab in three, brodalumab in two, and ixekizumab and golimumab in one each. Immunohistochemistry revealed high expression levels of S100A8 and S100A9, with less intense expression of S100A15. IL-36γ was detected in neutrophilic abscesses, as well as in the upper epidermis and cellular infiltrates below the epidermis. IL-36α and IL-36γ serum levels were elevated in the active phase of GPP compared to the inactive phase. In conclusion, this study highlights the systemic inflammatory nature of GPP, emphasizing its association with various comorbidities and organ involvement.

## Introduction

Pustular psoriasis is a rare subtype of psoriasis, classified into two primary forms: generalized and localized. Generalized pustular psoriasis (GPP) is a systemic disease characterized by widespread superficial sterile pustules affecting the trunk and extremities, which often rapidly develop into erythroderma. GPP is further divided into Zumbusch type, impetigo herpetiformis (acute GPP of pregnancy), annular and circinate forms, juvenile and infantile pustular psoriasis, and a generalized form of acrodermatitis continua of Hallopeau (ACH). In some cases, GPP patients present with pustular lesions on the palms, soles, and digits, which may resemble the clinical features of palmoplantar pustulosis (PPP) if confined to acral areas. By contrast, localized forms of pustular psoriasis include ACH and annular pustular psoriasis. ACH is a rare variant of pustular psoriasis that follows a chronic course, exclusively involving the distal phalanges and nails of the hands and feet. Annular pustular psoriasis, typically localized, can occasionally progress to a generalized form, accompanied by systemic manifestations, such as high fever, chills, and systemic inflammation. Several reports have been published on the clinicopathological analysis of pustular psoriasis, both within and outside Japan [1-4]. GPP, recently proposed as an autoinflammatory keratinization disease [5], is more frequent in Asian populations compared to Caucasian populations, suggesting a genetic predisposition in Asian populations. In the present study, we analyzed the clinical characteristics of 38 GPP cases diagnosed at our department over the last 15 years. Additionally, we examined local IL-36 expression and serum IL-36 levels in these cases.

## Materials and methods

Study design

We retrospectively collected data on age, gender, anatomical location, clinical variants, comorbidities, and therapeutic response for patients diagnosed with GPP at our university hospital between 2009 and 2023.

Study population and sample size

GPP was diagnosed in 38 patients based on the Japanese criteria: (a) systemic symptoms such as fever and fatigue, (b) generalized or extensive erythema, often accompanied by multiple sterile pustules that may coalesce to form lakes of pus, (c) histopathological features characterized by neutrophilic subcorneal pustules, specifically Kogoj’s spongiform pustules, and (d) recurrent episodes of generalized sterile pustulation [6]. All of our patients received a biopsy. PPP was excluded from the study.

Study measures

Using the biopsy samples, immunohistological staining was prepared by the standard avidin-biotin peroxidase technique with primary antibodies against IL-36γ (Serotec, Dusseldorf, Germany), S100A7 (Proteintech, Rosemont, IL, USA), S100A8 (Proteintech, Rosemont, IL, USA), and S100A15 (Bioss, Boston, MA, USA). Subsequently, the sections were incubated with Envision+Dual Link System-Horseradish Peroxidase (Dako Cytomation, Kyoto, Japan) or with biotinylated rabbit anti-rat IgG (Dako Cytomation). Antibody localization was determined using 3,30-diaminobenzidine (DAB, Nichirei Bioscience Inc., Tokyo, Japan). The sections were then counterstained with hematoxylin, dehydrated, cleared, and mounted. Negative controls were prepared by replacing the primary antibodies with non-specific rabbit IgG. Serum samples were obtained from GPP patients and stored at -80ºC. Serum levels of IL-36α and IL-36γ were measured using enzyme-linked immunosorbent assay (ELISA) kits (R&D Systems, Minneapolis, MN, USA), according to the manufacturer’s instructions.

Ethics statement

The Institutional Review Board of Fukushima Medical University approved this study (approval number: 30233).

Statistical analysis

Analyses were performed using Student’s t-test. All analyses were conducted using SPSS Statistics version 29 (IBM Corp. Released 2022. IBM SPSS Statistics for Windows, Version 29.0. Armonk, NY: IBM Corp.). All statistical tests were two-sided, and a p-value of < 0.05 was considered statistically significant.

## Results

Clinical features of the 38 patients with GPP

**Figure 1:**
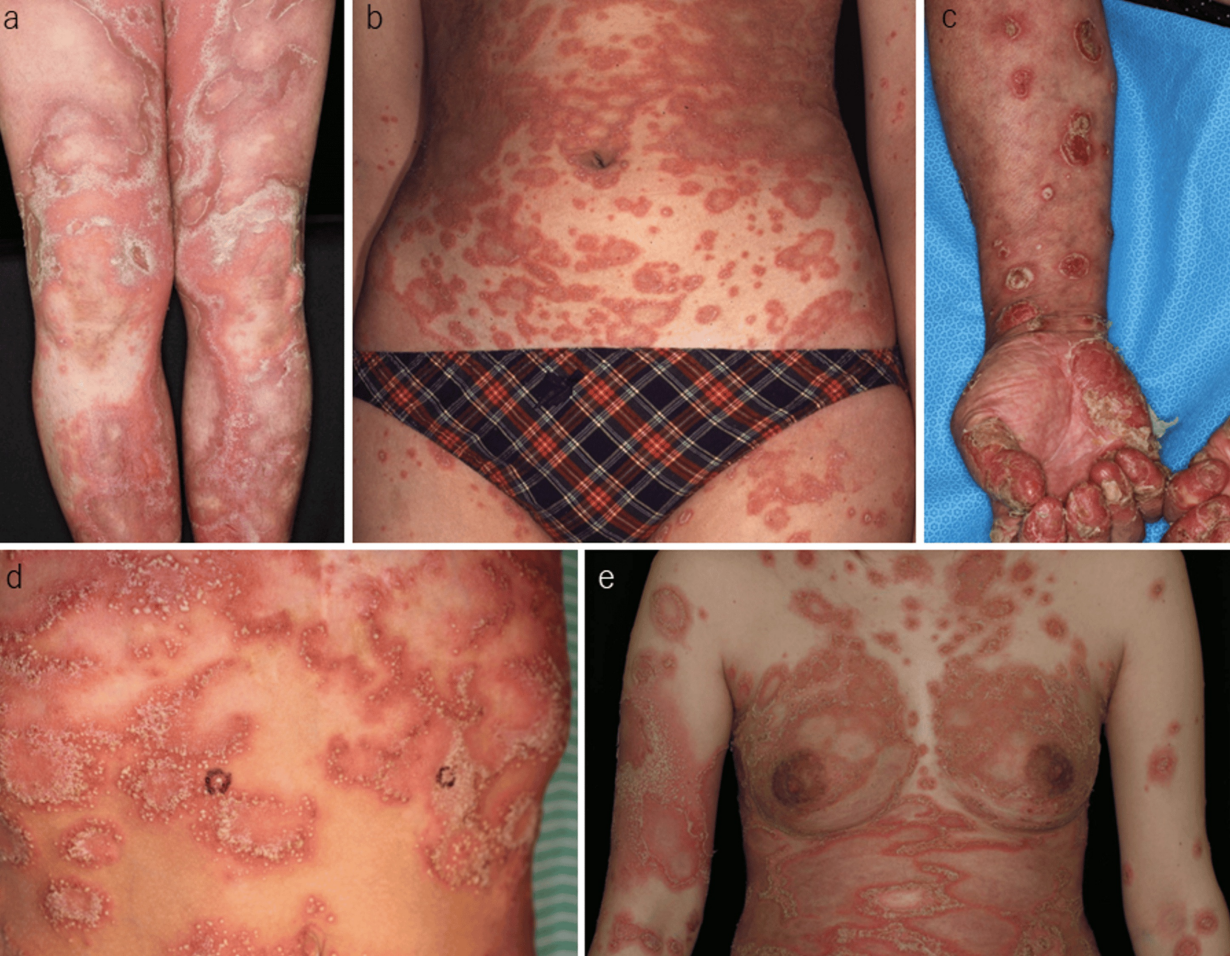
Clinical features of patients with GPP: (a) GPP without prior psoriasis (Zunbusch), (b) pediatric case, (c) GPP with prior psoriasis, (d) childhood GPP with Turner syndrome, and (e) impetigo herpetiformis GPP: generalized pustular psoriasis

**Figure 2:**
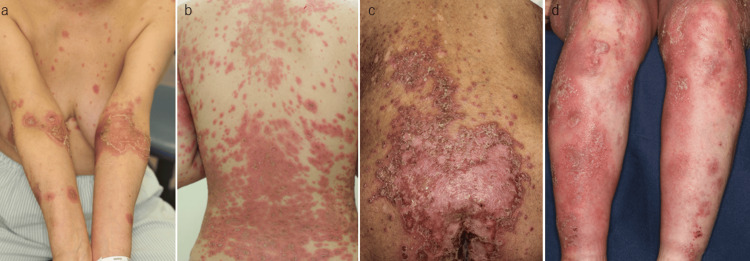
Clinical manifestations of GPP triggered by oral prednisone (a), terbinafine (b), sunitinib (c), and vaccination (d) GPP: generalized pustular psoriasis

GPP was observed in 38 patients, consisting of 20 males and 18 females. In contrast, localized pustular psoriasis was observed in three patients (two with annular pustular psoriasis and one with ACH). The onset age of GPP ranged from three months to 94 years old (median, 52 years). Among the 38 patients, 15 (39.5%) had a prior history of psoriasis, while the remaining patients developed GPP de novo. Pediatric onset was observed in four patients, including one with Turner syndrome. Representative clinical images are shown in Figure 1. Episodes of generalized pustulation occurred once in three patients, twice in three, and three times in one. The triggers of pustulation were upper respiratory infection (n=2), pregnancy (n=2), withdrawal of methotrexate (n=1), and infliximab (n=2) (Figure 2). Ten patients were former smokers, eight were current smokers, and 16 were never smokers (the smoking status of four of them was unknown). Arthritis was observed in 12 patients, among whom eight had a history of psoriasis and four had no history of psoriasis. The comorbidities were hypertension (n=10), diabetes mellitus (n=6), dyslipidemia (n=2), hyperuricemia (n=2), chronic kidney disease (n=2), ischemic heart disease (n=4), Crohn’s disease (n=3), and acute respiratory distress syndrome (n=1). Nail involvement was observed in 21 patients. Regarding treatment, etretinate was administered to 20 patients, cyclosporine to nine patients, methotrexate to two, and apremilast to one. Biologics were used in 14 patients: infliximab in eight, adalimumab in three, secukinumab in six, brodalumab in two, ixekizumab in one, and golimumab in one. The characterization of the 38 patients is shown in Table 1.

**Table 1:** Clinical characteristics of patients with pustular psoriasis * Student's t-test GPP: generalized pustular psoriasis

	Localized pustular psoriasis	GPP		
	(n=3)	(n=38)		
		With preceding psoriasis	Without preceding psoriasis	P
		(n=15)	(n=23)	
Male-to-female ratio	1:2	9:6	11:12	0.52
Mean age (yrs)	58.3	57.8	53.0	0.81
Mean onset age (yrs)	46.3	54.9	45.9	0.70
Pediatric onset	0	0	3	0.25
Weight (kg)	40.6（n=1)	60.2	57.4	0.41
Mean BMI	28.9（n=1)	23.1	23.0	0.48
Family history	0	1	0	0.40
Number of attacks	0	2	5	0.61
Smoker	0	10	8	0.10
Nail involvement (%)	1	5	16	0.05^*^
Arthralgia	0	8	4	0.03^*^
Comorbidities				
Hypertension	1	3	7	0.71
Diabetes mellitus	1	2	4	1.00
Hyperlipidemia	1	1	1	1.00
Hyperuricemia	0	2	0	0.15
Chronic kidney disease	0	1	1	1.00
Cardiovascular disease	0	1	3	1.00
Crohn’s disease	0	0	3	0.25
Acute respiratory distress syndrome	0	0	1	1.00
Systemic treatment				
Biologics	0	7	7	0.49
Etretinate	1	8	12	1.00
Methotrexate	0	1	1	1.00
Cyclosporine	0	3	6	1.00
Apremilast	0	1	0	0.40

Immunohistochemical analysis of S100A8, S100A9, S100A15, IL-36α, and IL-36γ in the lesional skin

**Figure 3:**
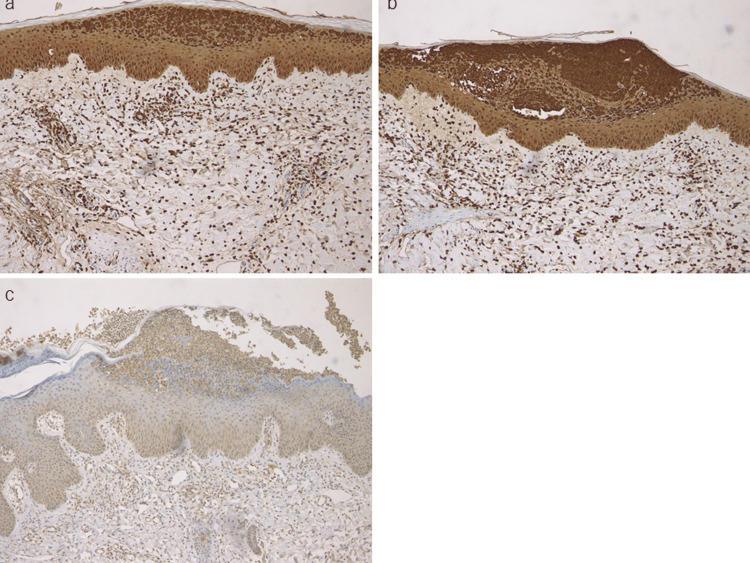
Expression of S100A8 (a), S100A9 (b), and S100A15 (c) in the lesioned skin of GPP GPP: generalized pustular psoriasis

**Figure 4:**
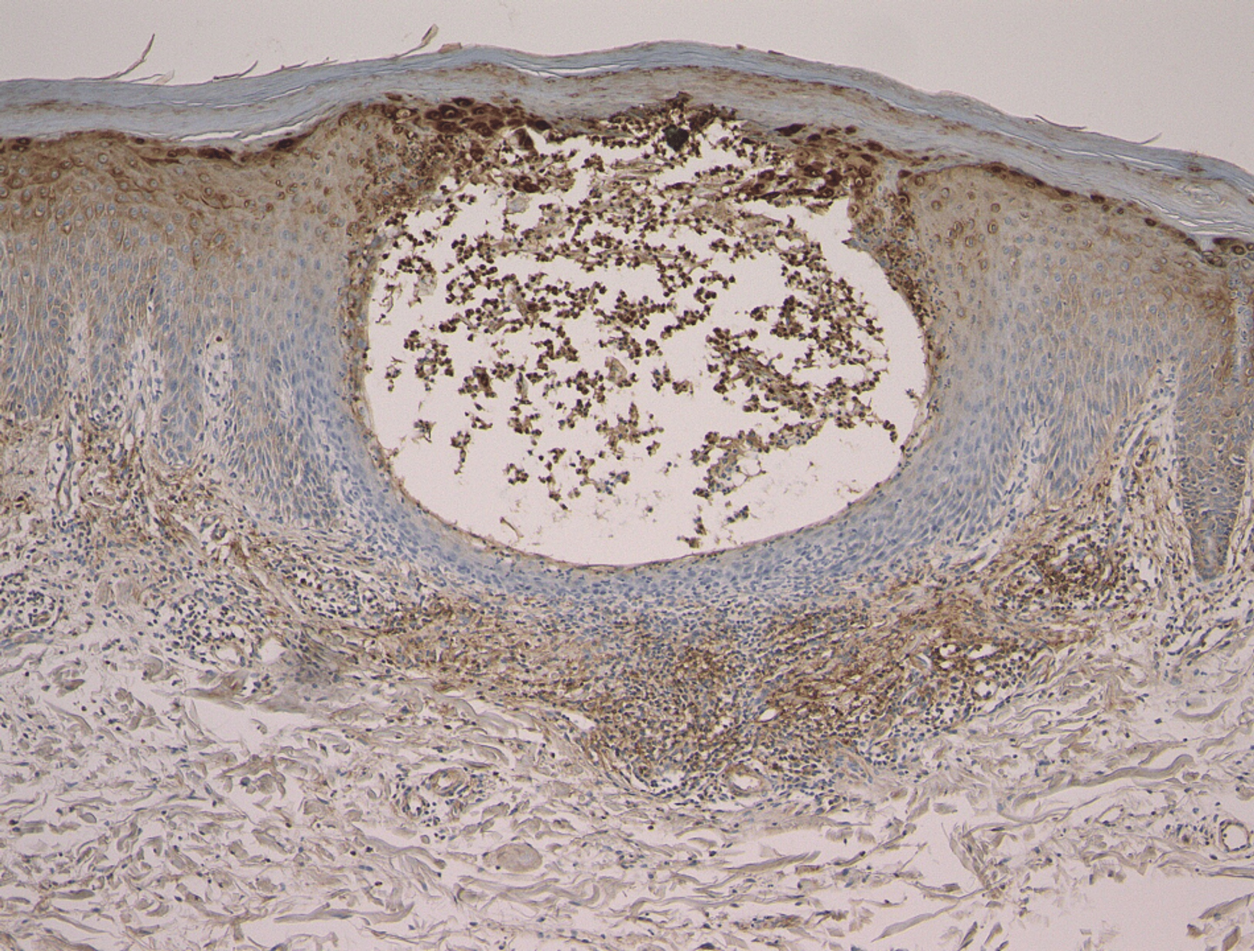
IL-36γ expression in the lesioned skin of GPP GPP: generalized pustular psoriasis

S100 proteins are expressed in a cell- and tissue-specific manner and are involved in various innate and adaptive immune responses, tissue development and repair, and the inflammatory process. Several S100 proteins, such as S100A8, S100A9, and S100A15, can serve as biomarkers in various inflammatory diseases, including psoriasis. In addition, recent studies implicate an essential role of IL-36 in GPP. Thus, immunohistochemical analysis of S100A8, S100A9, S100A15, IL-36α, and IL-36γ was performed in the lesional skin. Skin biopsies were carried out in all cases, revealing Kogoj’s spongiform abscess within the epidermis. Immunohistochemistry showed local expression of both S100A8 and S100A9 in the epidermis and infiltrating mononuclear cells in the upper dermis. Intense expression of S100A8 and S100A9 was observed in the epidermis and neutrophilic abscesses within the epidermis (Figure 3a-3b). By contrast, S100A15 was mildly stained in the lower layers of the epidermis (Figure 3c). IL-36γ expression was observed in the neutrophilic abscesses, upper layers of the epidermis, and cellular infiltrates below the epidermis (Figure 4).

ELISA assay of IL-36

Serum IL-36α levels in active GPP patients (n=21) (1311±2228 pg/ml) were significantly higher than those in inactive GPP patients (n=5) (30.0±5.4 pg/ml) (p<0.01, Student’s t-test). Similarly, IL-36γ levels in active patients (n=20) were higher (387±661 pg/ml) than those in inactive GPP patients (n=5) (35.6±11.4 pg/ml); however, the difference did not reach statistical significance.

## Discussion

Characteristics of Japanese patients with pustular psoriasis

In the present study, males were slightly higher than females, with a ratio of 10:9; however, Japanese patients with psoriasis vulgaris typically show a male predominance. Male patients have higher rates of smoking and alcohol consumption, and these lifestyle factors are suggested to contribute to an increased risk of psoriasis in men [7].

In contrast, previous reports from Korea showed a female predominance in pustular psoriasis [1]. The triggering factors were drugs, pregnancy, and infection, which were consistent with those previously reported; however, the causes were unknown in some patients. The reported percentage of patients with a history of plaque psoriasis varied between 10.7% and 42.4% [1]. Our results showed that GPP patients with a prior history of psoriasis vulgaris accounted for 39.5% (n=15). We compared the number of attacks (generalized pustulation) in the 38 patients with and without a history of psoriasis; however, no statistically significant difference was observed. The comorbidities were similarly observed between the 15 patients with and the 23 patients without a history of psoriasis.

Differential diagnosis

**Figure 5:**
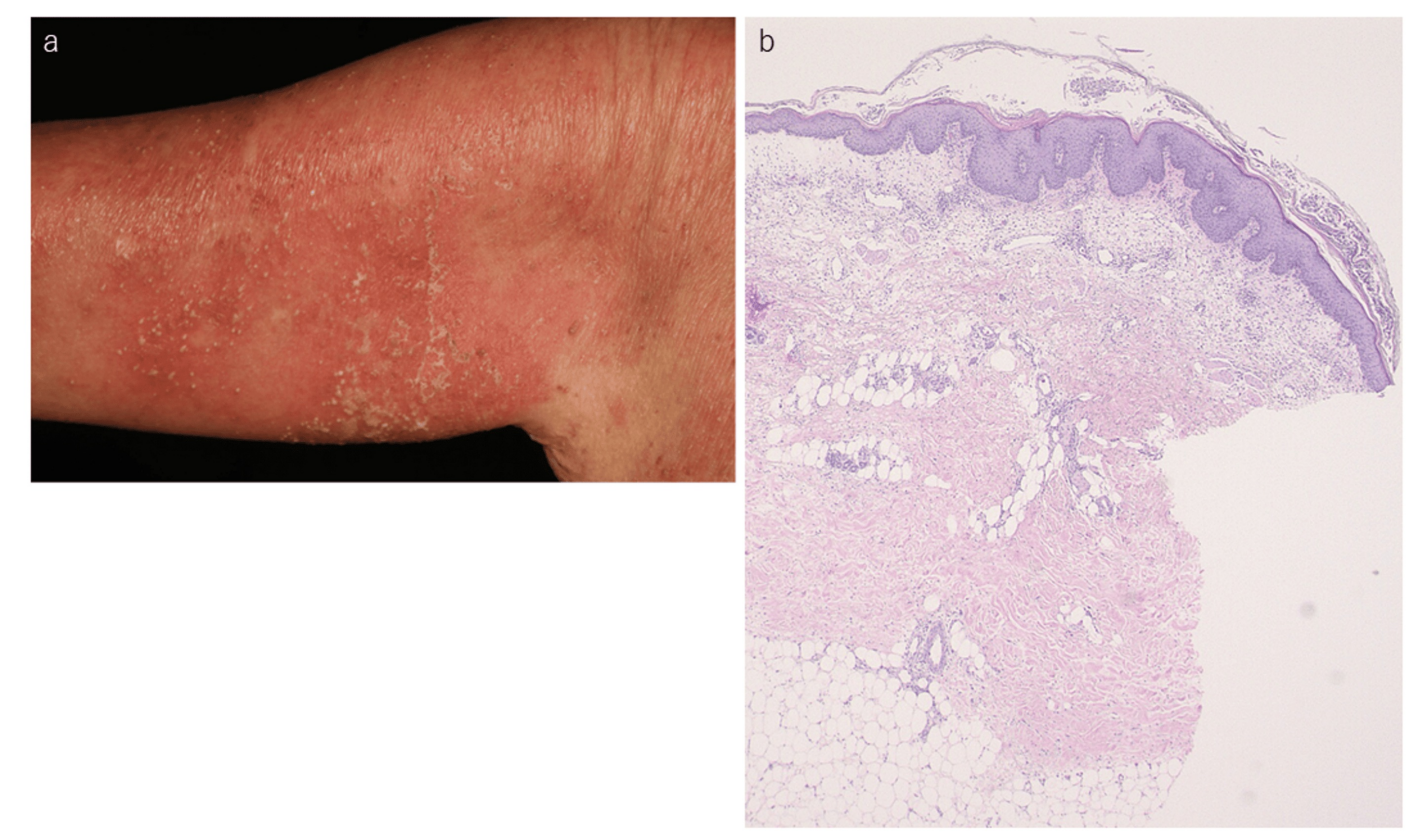
Acute generalized exanthema pustulosis mimicking GPP: (a) multiple small superficial pustules on the erythematous background and (b) biopsy showing subcorneal neutrophil infiltration without Kogoj spongiform abscess GPP: generalized pustular psoriasis

Recently, several diagnostic criteria for GPP have been proposed [8,9]. The differential diagnosis of GPP includes acute generalized exanthematous pustulosis (AGEP), IgA pemphigus, and subcorneal pustular dermatosis [10]. Among these, distinguishing AGEP poses the most significant challenge [11], particularly because AGEP can occasionally occur in patients with autoimmune diseases, including psoriasis. One representative case was a 70-year-old woman with a prior history of psoriasis, who developed generalized erythema accompanied by superficial small pustules (Figure 5a). A biopsy conducted due to suspicion of GPP showed no Kogoj’s spongiform abscess (Figure 5b), leading to the diagnosis of AEGP rather than GPP.

Comorbidities of pustular psoriasis

Representative comorbidities of psoriasis include cardiovascular diseases and metabolic syndrome, characterized by conditions such as obesity, hypertension, diabetes mellitus, insulin resistance, hyperlipidemia, and atherosclerotic diseases [12]. Patients with psoriasis are at an increased risk of developing metabolic syndrome, and those with severe, resistant psoriasis are significantly more likely to have metabolic syndrome compared with hospital-based controls. Recent large-scale studies from Japan reported that approximately 15-20% of GPP patients had diabetes, hypertension, and metabolic syndrome [1,13]. However, comprehensive data on a large cohort of GPP patients remains limited, and further research is needed to explore the role of adipokines in the pathogenesis of GPP. Additionally, while psoriatic patients have an increased risk of myocardial infarction, as well as cerebrovascular and peripheral vascular diseases, evidence specific to GPP patients is primarily confined to case reports. Pulmonary involvement in the course of psoriasis is considered to be extremely rare, with only a few cases of acute respiratory distress syndrome reported in association with GPP [12]. Possible etiologies include capillary leak syndrome, mediated by proinflammatory cytokines. Inflammatory bowel diseases such as ulcerative colitis and Crohn’s disease are sometimes complicated by psoriasis. Recent studies have demonstrated that IL-36 plays an essential role in intestinal immunity [14], suggesting a shared pathogenesis involving IL-36 between GPP and inflammatory bowel disease. In addition, neutrophilic cholangitis is rarely observed in association with GPP [15]. Patients with psoriasis have been reported to experience various ophthalmological complications, including blepharitis, conjunctivitis, keratitis, xerosis, symblepharon, trichiasis, uveitis, iridocyclitis, corneal ulceration, and corneal exfoliation. In particular, chronic uveitis has been reported to occur in association with pustular psoriasis occasionally. Tanaka et al. reported psoriatic uveitis in 13 Japanese patients, among whom three had pustular psoriasis [16].

Expression of alarmins

S100A8 and S100A9 are alarmins, also known as calprotectin. Expression of S100A8 and S100A9 is observed in psoriatic epidermis, whereas it is not in normal skin. Furthermore, S100A8 and S100A9 are products of neutrophils and monocytes, and the S100A8/A9 complex acts as an inflammatory cytokine. S100A15 (koebnerisin) is induced in cultured human keratinocytes upon stimulation with TNF-α, IFN-γ, and IL-1β [17]. S100A15 is directly linked to the innate immune system and is induced in response to injury, infection, and inflammation.

IL-36 is induced and released from epithelial cells as an alarmin, playing a role as a broad sensor of pathogenic damage or infection. IL-36 can be secreted by macrophages/monocytes, T cells, neutrophils, dendritic cells, and plasma cells as well. IL-36α, IL-36γ, and IL-36 receptor antagonist (IL-36RA) are mainly produced by keratinocytes after stimulation with inflammatory cytokines or toll-like receptor-3 ligands. Previous studies have shown that IL-36g expression in psoriatic epidermis was primarily localized from the suprabasal layer to the granular layer, with minimal expression in the basal layer [18,19]. In line with these findings, our results demonstrated that IL-36γ was primarily detected in the upper layers of the epidermis, in neutrophilic abscesses within the epidermis, and cellular infiltrates below the epidermis. The IL-1/IL-36-chemokine neutrophil axis is suggested as a core molecular pathway in the pathogenesis of pustular psoriasis [18,20,21]. Expression of IL-36α, IL-36γ, and IL-36RA correlates with disease activity and the levels of IL-1β and Th17 cytokines. IL-17 regulates IL-36 expression, and in turn, IL-36 potentiates the function of IL-17 [22]. Additionally, IL-36 signaling is a potent inducer of IL-23 [23]. IL-36 cytokines are activated by proteolytic cleavage by enzymes such as cathepsin G, elastase, and proteinase-3. In GPP, IL-36 cytokines can stimulate keratinocytes to produce chemokines that recruit neutrophils [18]. Thus, IL-36 plays a crucial role in the pathogenesis of GPP, and biologics targeting IL-36 are expected to provide beneficial effects in cases of refractory GPP [24,25].

GPP is a systemic inflammatory disorder

GPP is a systemic disorder affecting various organs [5,26] and is also associated with a significant disease burden and unmet medical needs. In the present study, patients were initially treated with several biologics, including infliximab, adalimumab, secukinumab, and brodalumab. However, these treatments required switching in multiple patients due to inefficacy, secondary failure, or adverse events. Of these six patients, five used two biologics and one used three biologics. Although we have previously used biologics other than IL-36 inhibitors, the biologics targeting IL-36 are currently available and may be further recommended as a helpful treatment option for GPP.

We have recently explored possible biomarkers for GPP and demonstrated that serum amyloid A (SAA) and leucine-rich α2-glycoprotein are expected as promising biomarkers of disease activity [27,28]. The serum levels of both mediators correlate with disease activity in GPP and increase during the active phase of the disease. SAA reflects inflammation, which may be caused by locally and/or systematically produced IL-1β and IL-6, as well as IL-36 [29]. Additionally, S100A8, A9, and A15 may be upregulated in response to the inflammatory milieu in pustular psoriasis. In the current study, immunohistochemistry revealed the expression of both S100A8 and S100A9 in the epidermis, as well as mononuclear cell infiltration in the upper dermis, suggesting that mechanical stimuli induce the expression of S100A8 and S100A9.

The present study has several limitations. First, it was a retrospective study conducted in a single institution. Second, while IL36RN mutations have been reported in GPP patients [30], mutation analysis was only performed in three cases, in which IL36RN mutations were detected in two cases, and one case yielded a negative result. Lastly, changes in IL-36 levels after biologic treatment were not examined. Despite these limitations, we believe that our analysis of 38 Japanese cases over an extended period provides valuable insights into the clinical characteristics of patients with pustular psoriasis.

## Conclusions

Our study suggested clinical variations of Japanese patients with GPP. This study highlights the systemic inflammatory nature of GPP, emphasizing its association with various comorbidities and organ involvement. Further studies are necessary to determine the appropriate treatment for elderly GPP patients with various comorbidities, as well as a personalized therapeutic approach for patients with or without an IL-36RN mutation.
